# Research on Optimization of Pooling System and Its Application in Drug Supply Chain Based on Big Data Analysis

**DOI:** 10.1155/2017/1503298

**Published:** 2017-02-15

**Authors:** DengFeng Wu, Hongyi Mao

**Affiliations:** Economics and Management School, Jiujiang University, Jiujiang 332005, China

## Abstract

Reform of drug procurement is being extensively implemented and expanded in China, especially in today's big data environment. However, the pattern of supply mode innovation lags behind procurement improvement. Problems in financial strain and supply break frequently occur, which affect the stability of drug supply. Drug Pooling System is proposed and applied in a few pilot cities to resolve these problems. From the perspective of supply chain, this study analyzes the process of setting important parameters and sets out the tasks of involved parties in a pooling system according to the issues identified in the pilot run. The approach is based on big data analysis and simulation using system dynamic theory and modeling of Vensim software to optimize system performance. This study proposes a theoretical framework to resolve problems and attempts to provide a valuable reference for future application of pooling systems.

## 1. Introduction

China has a population of more than 1.3 billion, which is one-fifth of the world population. In the past 30 years, the Chinese government has been pushing reform in the medical care industry to address the increasing demands of citizens for economic development [1]. The use of Internet and mobile technology significantly changes the way of doing business in healthcare industry [2–4]. For example, mobiles become a useful tool in searching health care information, including drugs, doctors, hospital, and treatments [5]. A lot of money has spent on IT projects to improve the service level of health care [6–8]. In 1994, the government started its reform in medical insurance and payment system in two pilot cities, namely, Jiujiang of Jiangxi province and Zhenjiang of Jiangsu province. The argument and exploration regarding medical care reform never ceased since then.

A Centralized Drug Procurement System was implemented in many provinces. This system requires drug suppliers to attend bidding, and only those who win the bidding are provided with the opportunity to sell drugs at the bidding price to public hospitals in the province. In this way, the selection of drug suppliers changes and becomes multiobjective [9, 10]. This policy aims to reduce price and eliminate corruption in drug procurement. The continuous use of the system generates a large amount of data which is updated every second, forming a complex and difficult environment for the health care reform in every aspect [11, 12]. This environment will bring extra issues for operations in health care reform [13]. Both formal and informal control should be conducted to increase the system performance [14, 15].

Reform means risk, existing in every project [15–17], which also makes sense to the reform project in drug procurement. Two serious issues emerged based on the feedback of parties involved in the drug reform in this big data environment.

1. Liquidity is increasingly being stressed on suppliers or distributors. According to the survey, the amounts receivable of suppliers in only one province can exceed 1 billion RMB, which is approximately equivalent to 160 million USD. Thus, some parties have to delay payment to relieve liquidity stress.
2. The possibility of drug shortage in public hospitals has increased. Average rate can reach 20% and occasionally 50%.

The problems mentioned have led to negative impact on drug supply chain and patients. These problems are attributed to the lack of innovation in the supply mode to correspond to the reformed procurement mode. Thus, from the perspective of supply chain, optimizing and adjusting the supply mode are critical to the success of drug procurement reform.

Initially, the project team proposed the use of Drug Inventory Pooling System, which was applied to a number of pilot hospitals and suppliers in Jiujiang City, Jiangxi Province [1, 18]. At present, Jiujiang University Hospital sponsors four public and one community hospital. One supplier also joined the project.

Feedback for the first eight months of pilot run indicates that the average rate of drug shortage may reach 13%, which is better than the previous rate but worse than expectation. Suppliers expected progress after addressing the liquidity problem, but they remained stressed.

The Inventory Pooling System is an effective method of improving the drug supply chain, but a number of detailed problems should be optimized based on previous research [1, 19]. To address the problems identified in the pilot run and examine the deficiency of previous research, the present study collects big data from hospital. Based on big data of drug daily demand in hospital, a model is established to simulate variables' change in pooling system and provide solutions. This study is expected to provide a theoretical basis for the optimization of drug supply chain.

## 2. Literature Review

The surrounding conditions used in existing literature on Chinese medical care reform differ from those in other countries. However, we can still find papers that focused on the healthcare industry by employing System Dynamics (SD).

Chahal [20] discussed the process of establishing a hybrid simulation model through SD and discrete event simulation (DES) to resolve problems in the healthcare context.

Mehrjerdi [21] presented a SD model to study interconnections among human being weight, eating habits, exercise, body fat, medication intake, drugs used, and the health problems.

Jia et al. [22] established a model to demonstrate the in-depth mechanism of the influence of medicine price policy on drug prescription; this study also proposed countermeasures.

Smith III and Roberts [23] presented a hospital's simulation model of SD to explore the influence of interacting unit capacities on overall efficiency and performance. This study showed the important factors that significantly contribute to the overall performance and efficiency of a hospital enterprise. Based on the SD model, this study applied the sequential bifurcation technique to identify important factors.

Utami et al. [24] focused on the supply chain contract as a dynamic process of cooperative interorganization relationships in the pharmaceutical industry in Indonesia. They aimed to develop a SD model for contracts in the pharmaceutical supply chain by considering price and demand risks to provide alternative policies for an equitable supply chain contract for all parties in the pharmaceutical supply chain.

Behzad et al. [25] established a model and simulated the internal service supply chains of a healthcare system to study the effects of different parameters on the outputs and capability measures of processes.

The above literature indicates that establishing a simulation model based on SD is a good method to solve problems in a drug supply chain. Good modeling through SD theory is based on big data collection. Effective and costly data collection can help improve the effect and accuracy of simulation.

## 3. Establishing a Simulation Model

Everything in this world is a component of a system [26]. The world we live in is a huge system. Human undergoes the process of exploring the world's structure, components, and interrelations, as well as the interaction of people to master the objective law of the world. People establish diverse mathematical models to describe the objective rules of development. A model is an effective tool for system analysis and system design. The use of mathematical models to conduct system simulation will facilitate the identification of existing problems and intuitively optimize the system [27–29].

### 3.1. Problems Raised

With regard to the drugs “Levofloxacin Hydrochloride and Sodium Chloride injection” in literature 1, the original SD model was established by Vensim software based on daily demand data of the drug in 2.5 years in three sample hospitals. That model contains the following problems.

*(1) The Influence of Key Variables in the Optimization of the Pooling System Has Not Been Completely Studied*. The original model was established in one chapter of the dissertation. Given the limited space in the dissertation, a few key variables of Inventory Pooling System, such as “purchasing leading time,” “replenish cycle,” and “service level of safety stock” and their influence on “liquidity occupied” and “drug shortage rate,” have not been discussed. Finding the best value of variables using the simulation model is necessary because adjusting those variables will directly affect the performance of the pooling system. 

*(2) The Relationship between Pooling System and a Number of Sample Hospitals Has Not Been Discussed*. The original model was subjected to a number of sample hospitals. Thus, data were based on only three hospitals. The amount of liquidity required and the change in drug shortage were not discussed when more than three hospitals are involved in a system. Traditionally, involvement of several hospitals will result in obvious scale effect of the pooling system. Simulation will help suggest accurate scale and range. 

**Figure 1:**
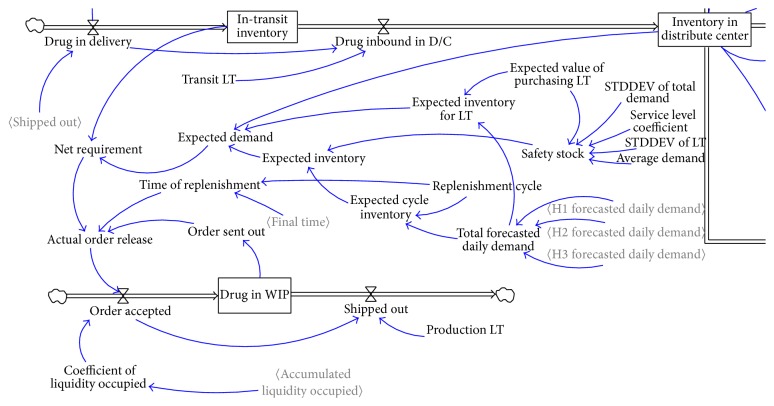
Simulation flow chart about process of drug purchasing in traditional model.

*(3) Calculation of Purchasing Volume Should Be Reasonable*. In the original model (Figure 1), reasoning about purchasing volume in distribution center is not reasonable. In the new model, procurement theory of P-system [30] and the process of maximizing demand volume to net requirement volume and placing order in procurement will be simulated and modeled. 

*(4) The Root Causes That Resulted in the Reduced Inventory Level in a Supply Chain and Its Improvement Were Not Discussed*. In a pooling system, inventory level reduction may be attributed to the following factors:

- Less procurement cycle and high frequency of placing an order.
- The pooling system has its own inherent advantage. Therefore, even in a similar situation, the application of pooling system will reduce inventory.

History literature, which is a primary factor in the preceding causes, has never been discussed. However, history literature can assist in finding solutions to optimize the pooling system. If the first item works, then improving the purchasing cycle and frequency can be a good option. If the second item works, then the next step should involve additional hospitals for improved optimization. 

*(5) Inadequate Reality Checks*. By taking Hospital H1 as example, the original model equation of drug distribution is set as

(1) drug  delivered  to  H1=MINMAXMAXH1  hospital  planned  order−H1  hospital  inventory,0,MAXH1  daily  demand  from  patient−H1  hospital  inventory,0,Inventory  in  Distribute  center.

**Figure 2:**
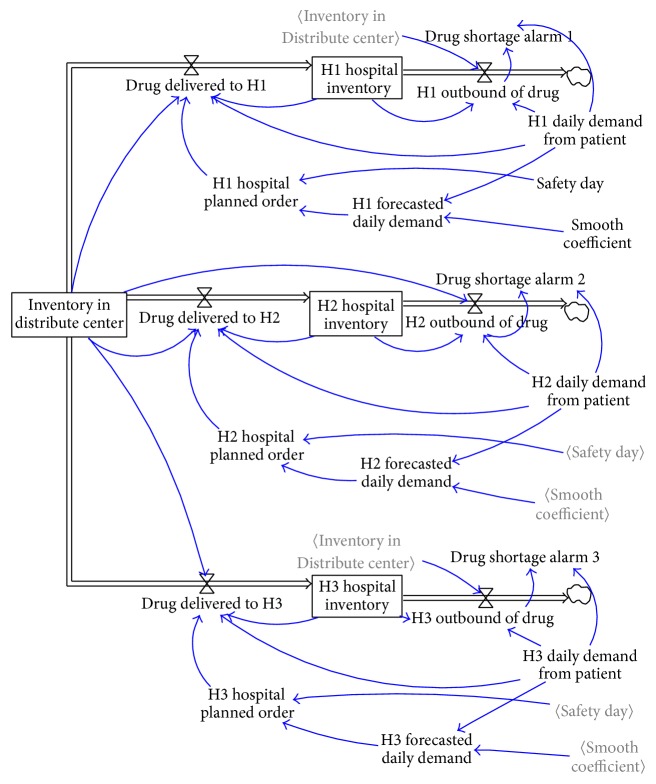
Simulation flow chart regarding drug delivered from D/C to the hospital in the old model.

**Figure 3:**
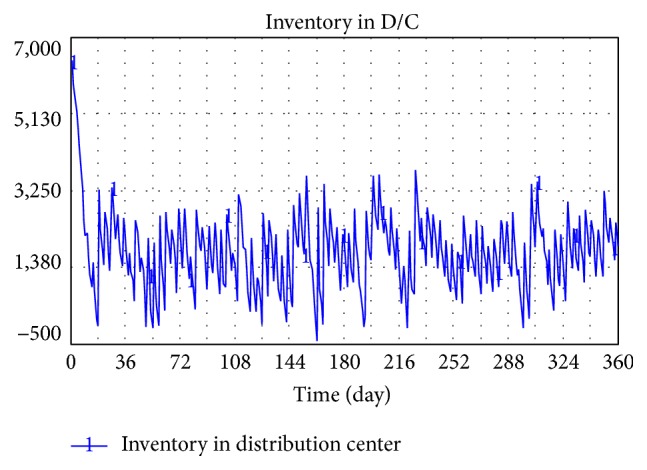
Simulation of inventory in D/C in a few situations in the original model.

The equation obtains a minimum value between hospital demand and inventory level in the distribution center (D/C) as delivered volume to ensure D/C inventory is not less than zero. When only one hospital has a demand, the result is considered true. However, in a pooling system, total demand from multihospitals is occasionally larger than D/C inventory though the demand of each hospital is smaller than D/C inventory (shown in Figure 2). In that case, D/C inventory will be negative and will result in simulation failure (shown in Figure 3).

### 3.2. Remodeling

**Figure 4:**
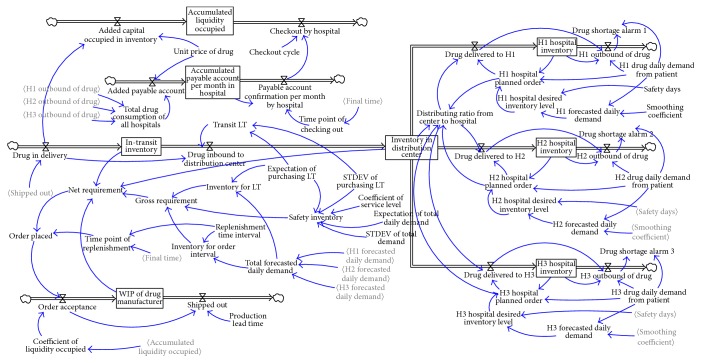
Simulation flow chart model after improvement.

Final time is 360 days and time step is one day in the new model. SD was established by Vensim software (Figure 4).

A few changes were introduced into the new model based on the original model in literature 1.

#### 3.2.1. Equation Setting for Nonnegative Number of D/C Inventory Level

The model added the variable “distributing ratio from center to hospital” to compare D/C inventory and the total demand of hospitals and increase inventory level in the simulation model to higher than zero. The division of these two factors is fulfillment rate.

(2) Distribution  ratio  from  center  to  hospital=IF  THEN  ELSEH1  hospital  planned  order+H2  hospital  planned  order+H3  hospital  planned  order>Inventory  in  distribution  center,MaxInventory  in  distribution  center,0H1  hospital  planned  order+H2  hospital  planned  order+H3  hospital  planned  order,1,drug  delivered  to  H1=H1  hospital  planned  order∗distributing  ratio  from  center  to  hospital.

**Figure 5:**
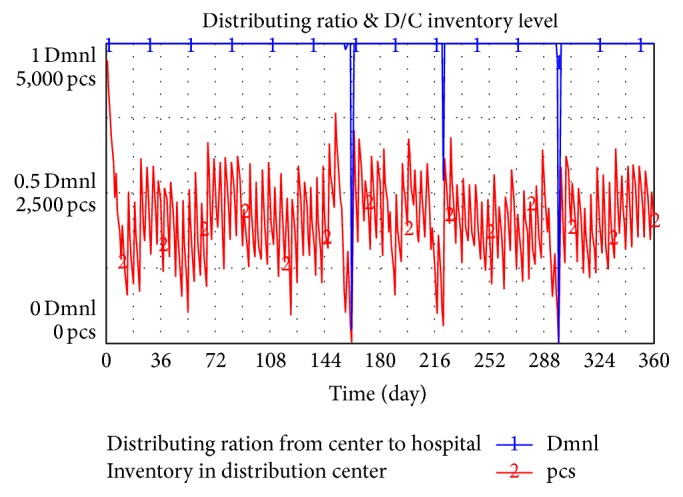
D/C inventory level and distributing ratio.

Figure 5 shows the result of simulation. The figure shows that inventory in D/C sometimes fails to meet demand, which may cause supply break. However, inventory level is always larger than or equal to zero.

#### 3.2.2. Optimize the Process of Placing Order

According to the classic P-system ordering theory, the relationship between factors in the new model is given as

(3) Gross  requirement=inventory  for  LT+inventory  for  order  interval+safety  inventory.

Net requirement is the gap between gross requirement and current inventory, which include inventory in transit, D/C, and WIP (work in process).

(4) Net  requirement=Maxgross  requirement−“In-transit  inventory”−Inventory  in  distribution  center−WIP  of  drug  manufacturer,0,Order  placed=net  requirement∗time  point  of  replenishment,Time  point  of  replenishment=PULSE  TRAIN0,1,REPLELISHMENT  TIME  INTERVAL,FINAL  TIME,here  REPLELISHMENT  TIME  INTERVAL  is  four  days.

#### 3.2.3. Modeling of Purchasing Leading Time (LT)

According to a survey, the expected value of lead time of Levofloxacin Hydrochloride and Sodium Chloride injection is seven days, whereas its standard deviation is three days. The entire purchasing L/T comprises the production and transit period. This study focuses on drug distribution supply chain without loss of generality. Production period is set as constant at three days. The transit period fits the normal distribution with its expected value of four days.

(5) TRANSIT  LT=random  normal0,10,EXPECTATION  OF  PURCHASING  LT−3,STDEV  OF  PURCHASING  LT,0.

**Figure 6:**
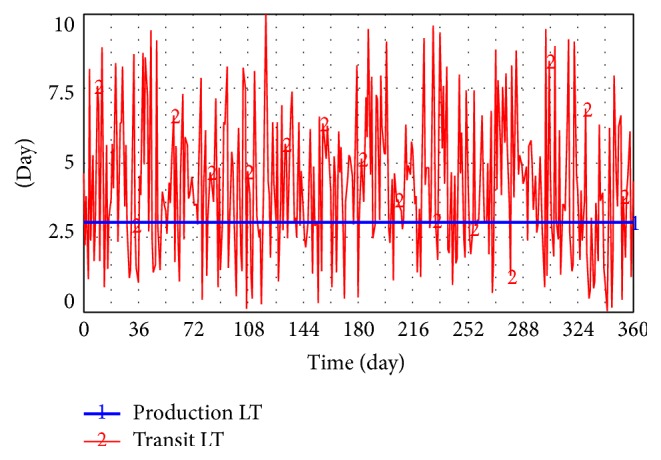
Simulation of Production LT and Transit LT.

In the preceding equation, the expected value of Transit LT is set as purchasing LT minus 3. Transit LT will synchronously vary when the expected value and STD deviation of purchasing LT are diminished (shown in Figure 6).

## 4. Inventory Pooling Optimized by Key Variables

Setting a few important variables and indexes in the pooling system will directly and obviously affect its effects [31, 32]. Based on the problems raised in background analysis, variables “accumulated liquidity occupied” and “drug shortage ratio” are selected as key indexes to measure the application effect of the pooling system. The definition of the former index is liquidity receivable but occupied in related links, such as in transit, D/C, and hospitals. The latter is the number of drug shortage times in a simulation life cycle.

### 4.1. Purchasing L/T and Replenish Cycle Adjustment

#### 4.1.1. Lead Time

In similar surroundings and parameters, purchasing L/T is separately set as 3, 9, 18, and 25 days to simulate the relationship between L/T and liquidity occupied.

**Figure 7:**
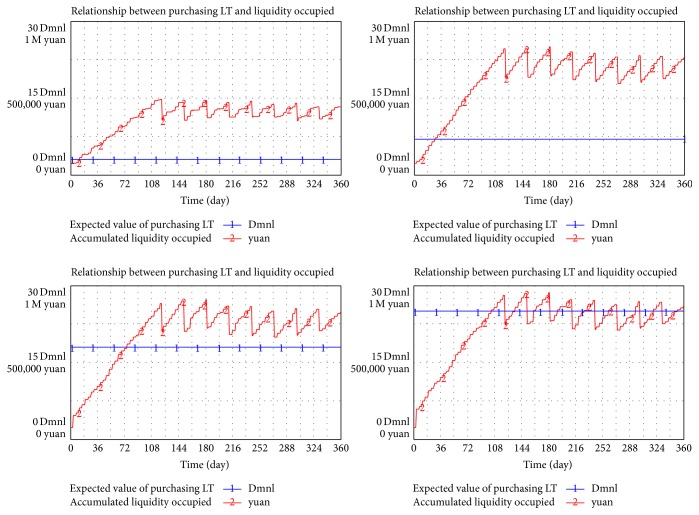
Relationship between purchasing LT and liquidity occupied.

In Figure 7, liquidity continuously increases (curve in red) with the increment of L/T (transverse line in blue). L/T has a proportional relationship with liquidity occupied in a supply chain; however, the relationship is not always linear. The increment rate of liquidity gradually slowed down. In summary, shortening L/T will benefit the reduction of liquidity occupied.

Without other changes in the surroundings and variables, L/T is separately set as 7, 5, 3, and 1 day to simulate its relationship with drug shortage.

**Figure 8:**
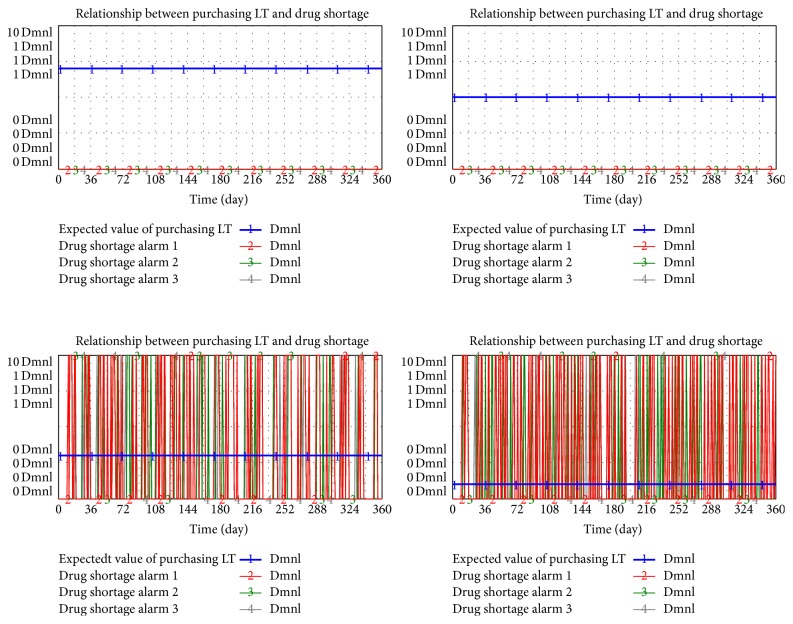
Relationship between purchasing LT and drug shortage.

In Figure 8, cutting down L/T (transverse line) will not increase the frequency of shortage (vertical line obviously). However, a relatively short L/D (such as 3 or 1 day in the figure) will result in a drastic increment of the shortage because a short L/D will lead to low-level safety stock. Once the random demand in the end of the supply chain drastically and unexpectedly fluctuates, the drug will be out of stock and supply breaks. In other words, pursuing a relatively short L/D is unnecessary for parties involved to reduce risk of shortage in a drug pooling system.

#### 4.1.2. Replenish Cycle

Replenish cycle is the interval between two consecutive order placements. In the same surrounding and other variables, the cycle time will be set as 2, 4, 10, and 25 days to simulate the change of liquidity occupied.

**Figure 9:**
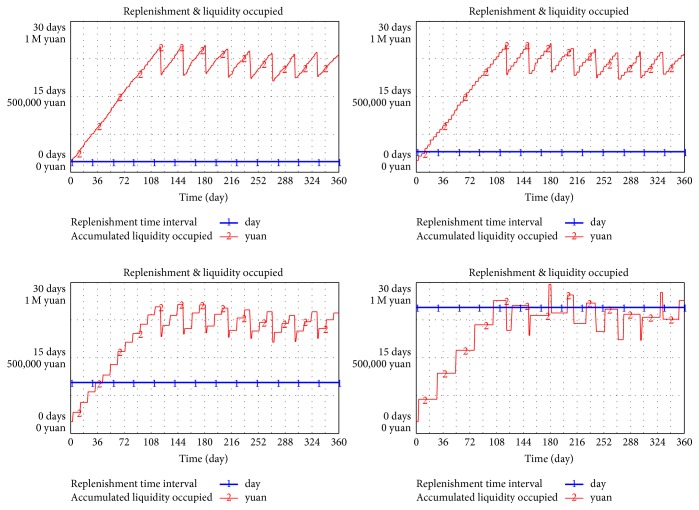
Relationship between replenishment cycle and liquidity.

In Figure 9, liquidity occupied (curve line) will slightly increase with the increment of replenish cycle (transverse line). In the simulation model, the corresponding average value is 620837, 626727, 644285, and 690170, whereas the maximum value is 824869, 838331, 852919, and 940703. The minimum value, which is the default value, is 98214. The root cause leading to this trend is as follows:

- Liquidity occupied is mainly influenced by the cycle of payment settlement.
- The maximum inventory in D/C comprises three parts, namely, inventory in replenish cycle, L/T, and safety stock. The increment of time cycle only affects the inventory in the replenish cycle but not either. Overall, the inventory is only slightly influenced.
- Annual total demand remains stable.

Other conditions remain unchanged. Replenishment cycle is set as 2, 4, 10, and 15 days to simulate the times of drug shortage.

**Figure 10:**
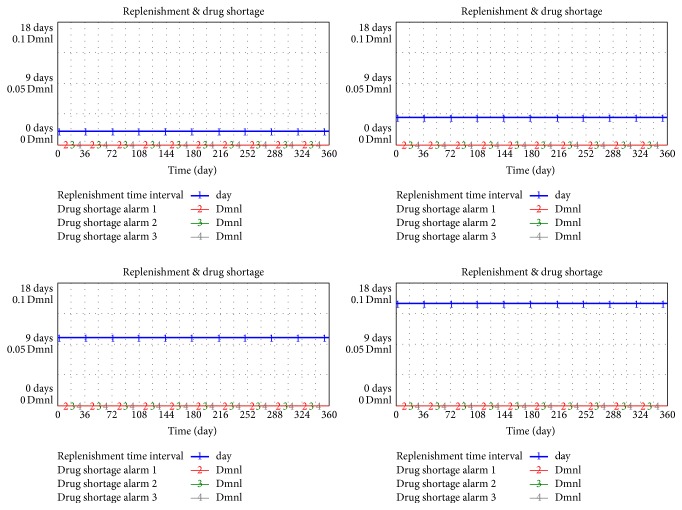
Relationship between replenishment cycle and drug shortage.

When cycle time (transverse line) increases, drug shortage frequency remains stable because drug shortage is only related to safety stock level instead of replenish cycle (shown in Figure 10). The latter decides the inventory in its own period.

### 4.2. Service Level

**Figure 11:**
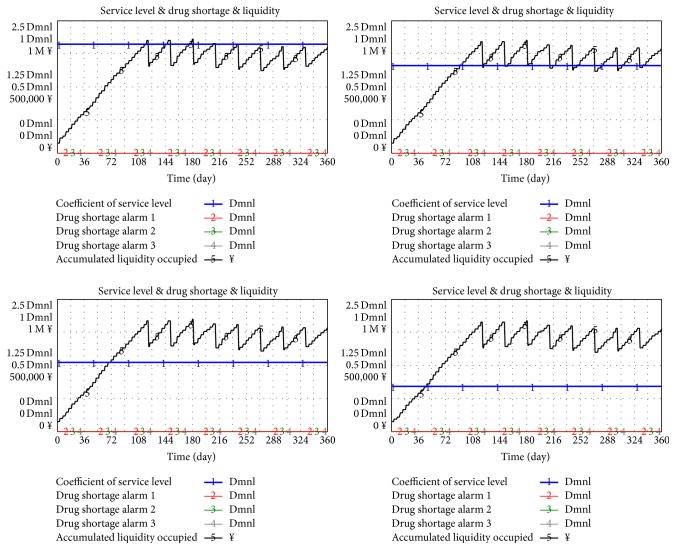
Relationship among service level, drug shortage, and liquidity occupied.

“Service level” describes the degree to which inventory addresses demand and is presented as a percentage. According to the Normal Distribution Chart, when the service level of safety stock is set as 98%, 95%, 90%, and 80%, the corresponding service coefficient will be 2.05, 1.65, 1.30, and 0.85, respectively. Once these values are placed in the formula, the relation between service level and KPI can be shown in Figure 11.

Service level has no obvious effect on KPI. Based on the details of the simulation data, when the service level changed from 98% to 90%, the average inventory level of the supply chain dropped to 4730, which achieved 17.32% improvement compared with 5720 (98%). Before service level changed to 90%, the frequency of drug shortage remained zero even when below 90%. Thus, drug shortage has never occurred.

### 4.3. Analysis of Additional Hospital Involvement in the Pooling System

Existing studies are based on the situation of three sample hospitals involved in the pooling system based on data in early investigation. The process of increasing KPI if additional hospitals join the system is discussed.

The involvement of additional hospitals should have the same possibility distribution with the three sample hospitals in a daily demand because all data were obtained from three hospitals based on the early investigation for easy comparison without loss of generality.

**Figure 12:**
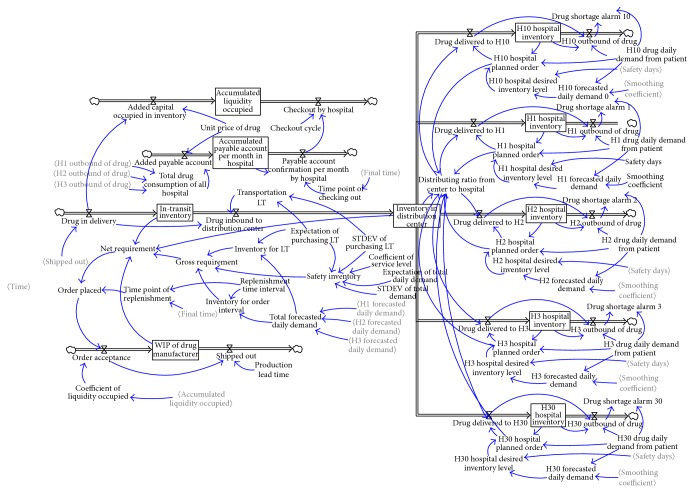
Simulation of five hospitals involved in the Inventory Pooling System.

In the new model, similar data features, such as expected value and standard deviation of daily demand as number 1 and number 3 hospitals and number 10 and number 30 hospitals respectively, are involved in the pooling system. The flow chart of the pooling system of five hospitals is shown in Figure 12 to save space.

**Figure 13:**
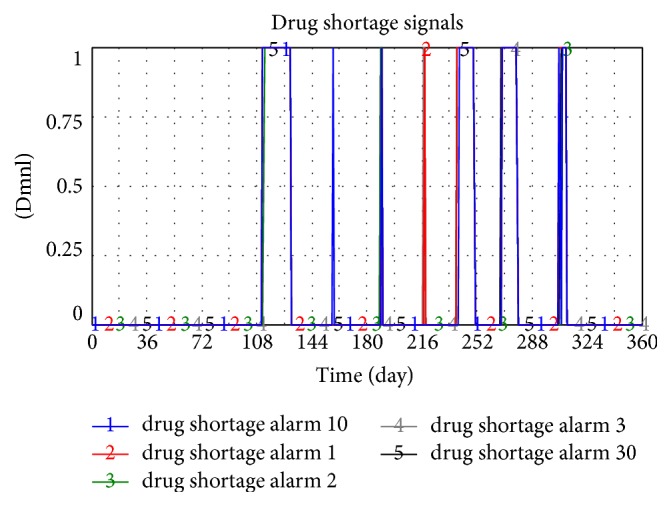
Drug shortage when five hospitals join the pooling system.

The simulation result shows that the total frequency of drug shortage occurred 234 times after five hospitals were involved (shown in Figure 13), which indicates 47 times for one hospital on average and the shortage rate is approximately 13% in 360 days in one year.

The main reason attributed to drug shortage is that suppliers need to address increased hospital demands and liquidity required rapidly increased thereby resulting in liquidity rupture and supply break.

Based on the analysis, the following measures can be applied to relieve stress of liquidity required: (a) finance increased liquidity; (b) accelerate rotation of liquidity.

According to government policy, hospitals should settle their accounts with suppliers in 90 days only after obtaining drugs. However, in the Centralized Drug Procurement System, a supplier winning a bid may gain an opportunity to provide drugs to all public hospitals in a provincial scope, which will lead to an enormous supply. Once the 90-day cycle of payment is completed, liquidity may be at risk of rupture. The trend attributed to the shortening payment cycle should be discussed.

In unchanged conditions, the set cycle of payment is 90, 60, 30, and 0 days (0 day means to pay by the end of the month).

**Figure 14:**
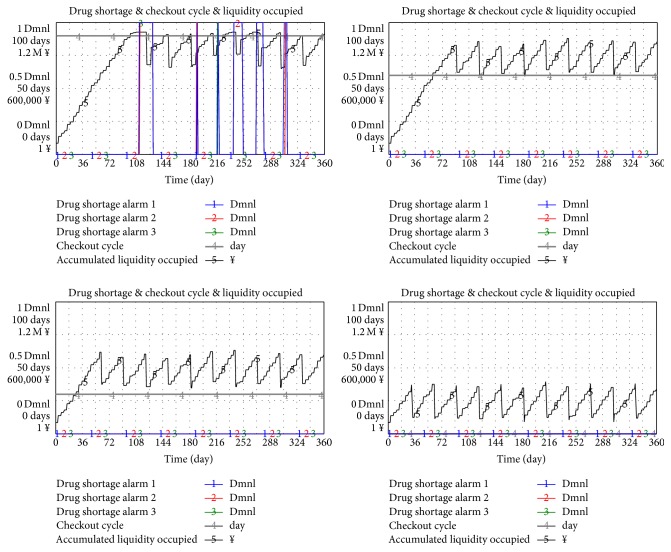
Relationship between checkout cycle and KPI when five hospitals are involved in a pooling system.

Figure 14 shows that the liquidity stress and shortage rate improved with the shortened payment cycle. When the payment cycle is set at 60 days or below, the shortage has never occurred again in three hospitals.

## 5. How Does the Pooling System Improve KPI

Traditionally, a pooling system has a natural advantage in cutting down the inventory level in a supply chain. However, this study shows that inventory drop is caused by the shortened replenish cycle.

In literature 1, the replenishment cycle in a traditional model is 30 days. Replenishment cycle improved to 30 days in the pooling system without any other change.

**Figure 15:**
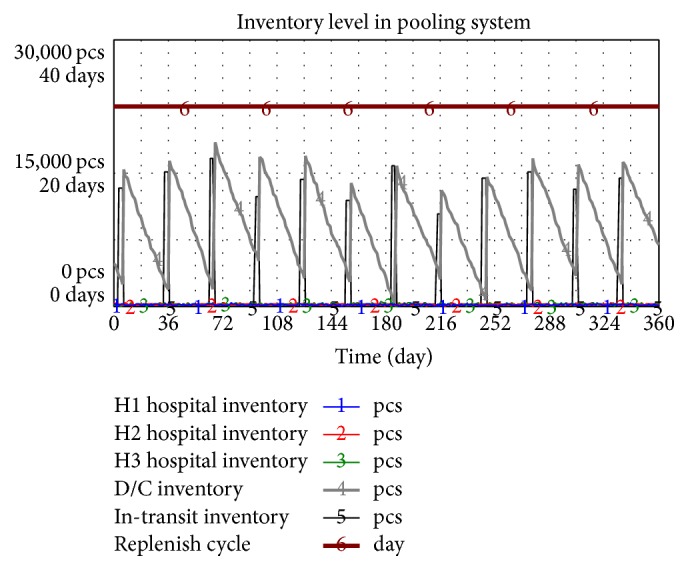
Inventory of the entire supply chain of pooling system.

Figure 15 shows that the total inventory of the entire drug supply chain in the pooling system is 10952. Inventory in three hospitals, inventory in D/C, and in-transit inventory are 124, 148, 192, 9112, and 1382.

**Figure 16:**
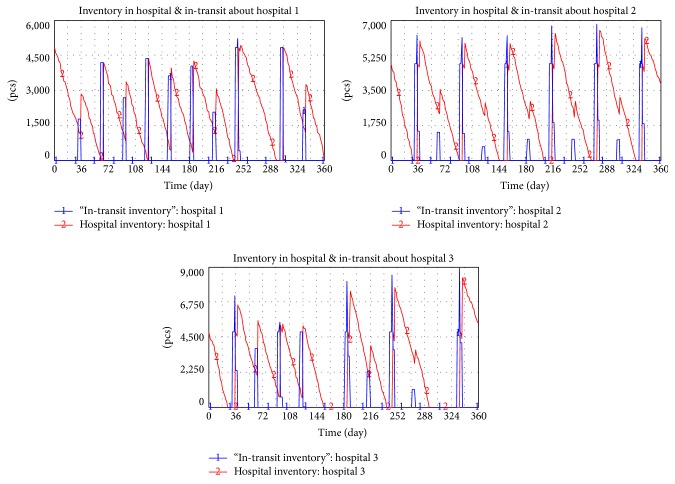
Inventory level in the traditional drug procurement model.

In literature 1, replenish cycle is 30 days in a traditional procurement model. Inventory in three hospitals and transit is shown in Figure 16. The entire supply chain of three hospitals is accumulated to 9178.

The replenish cycle of the pooling system to be applied will be promoted to 30 days. The average inventory level in a supply chain of drug distribution is 11649. Inventory in three hospitals, inventory in D/C, and in-transit inventory are 124, 148, 192, 9823, and 1362. The annual average inventory of the distribution supply chain is 5778 to shorten the replenish cycle to four days.

Analysis indicates that the pooling system cannot directly and obviously reduce inventory in a supply chain. However, given that the pooling system is implemented by a professional Third Party Logistics (TPL) instead of the hospital, frequent and small-scale purchase and distribution become possible. Inventory level will drop with purchasing scale reduction. Cutting down inventory cannot improve the status of liquidity stress because the latter is mainly influenced by payment cycle.

## 6. Conclusion

A few principles should be emphasized after the application of the pooling system to relieve stress of liquidity and reduce the frequency of drug shortage.

*(1) Shortening Payment Cycle to Reduce Liquidity Occupied*. After a government-oriented bidding system is applied in the drug purchasing industry, a supplier who wins the bid will gain the opportunity to supply all hospitals in one provincial scope. This process may result in a sharper supply volume surge than in history data and liquidity stress. Given the shortened payment cycle, liquidity amount will considerably decline because payment cycle can affect the amount of liquidity. Thus, when creating centralized purchasing laws, the government should require a short payment cycle to streamline the supply chain. 

*(2) Suppliers Should Shorten Replenishment Cycle to Cut down the Inventory Level*. The replenish cycle should be shortened to reduce inventory. Otherwise, the payment cycle should be shortened to relieve liquidity stress. 

*(3) Suppliers Should Not Set a Relatively Short Leading Time of Procurement*. Traditionally, short lead time is good for low-level inventory, good liquidity status, and quick response to the market. However, as per simulation, a relatively short leading time will also increase the risk of shortage. The supplier should set a reasonable lead time to balance different influences from L/T. 

*(4) Service Level of Safety Stock Should Be Proper*. A considerably high service level of safety stock will reduce the risk of drug shortage but increase the liquidity and inventory level. Once liquidity rupture occurs, consecutive supplies will be seriously influenced. Simulation achieves 90% improvement in service level. 

*(5) Supplier Should Set Reasonable Number of D/C*. The number of hospitals wherein one D/C responds to the pooling system will not benefit the reduction of inventory level and the improvement of liquidity in the supply chain. Designing a distribution network is only determined by the cost of building a warehouse, overhead, and distribution.

In summary, the drug pooling system is a systematic program. In its application, involved parties, such as the government, the supplier, and the hospital, should take good measure and set proper parameters to optimize inventory level and liquidity status. The new mode of drug supply chain can then address initial expectations.
